# The promoter of filamentation (POF1) protein from *Saccharomyces cerevisiae *is an ATPase involved in the protein quality control process

**DOI:** 10.1186/1471-2180-11-268

**Published:** 2011-12-28

**Authors:** Iris M Costa, Tallybia HT Nasser, Marilene Demasi, Rafaella MP Nascimento, Luis ES Netto, Sayuri Miyamoto, Fernanda M Prado, Gisele Monteiro

**Affiliations:** 1 Departamento de Tecnologia Bioquímico-Farmacêutica, Faculdade de Ciências Farmacêuticas, Universidade de São Paulo - USP, São Paulo-SP, Brazil; 2 Laboratório de Bioquímica e Biofísica, Instituto Butantan, São Paulo-SP, Brazil; 3 Departamento de Genética e Biologia Evolutiva, Instituto de Biociências, Universidade de São Paulo - USP, São Paulo-SP, Brazil; 4 Departamento de Bioquímica, Instituto de Química, Universidade de São Paulo - USP, São Paulo-SP, Brazil

**Keywords:** unfolded protein response, endoplasmic reticulum stress, antioxidant response

## Abstract

**Background:**

The gene *YCL047C*, which has been renamed promoter of filamentation gene (*POF1*), has recently been described as a cell component involved in yeast filamentous growth. The objective of this work is to understand the molecular and biological function of this gene.

**Results:**

Here, we report that the protein encoded by the *POF1 *gene, Pof1p, is an ATPase that may be part of the *Saccharomyces cerevisiae *protein quality control pathway. According to the results, *Δpof1 *cells showed increased sensitivity to hydrogen peroxide, *tert*-butyl hydroperoxide, heat shock and protein unfolding agents, such as dithiothreitol and tunicamycin. Besides, the overexpression of *POF1 *suppressed the sensitivity of *Δpct1*, a strain that lacks a gene that encodes a phosphocholine cytidylyltransferase, to heat shock. *In vitro *analysis showed, however, that the purified Pof1p enzyme had no cytidylyltransferase activity but does have ATPase activity, with catalytic efficiency comparable to other ATPases involved in endoplasmic reticulum-associated degradation of proteins (ERAD). Supporting these findings, *co-immunoprecipitation *experiments showed a physical interaction between Pof1p and Ubc7p (an ubiquitin conjugating enzyme) *in vivo*.

**Conclusions:**

Taken together, the results strongly suggest that the biological function of Pof1p is related to the regulation of protein degradation.

## Background

Cells possess several mechanisms to control the quality of their components, such as proteins [1]. One of these mechanisms ensures proper folding and function of proteins, sending misfolded proteins to be degraded by the ubiquitin-proteasome system and represents the best characterized protein quality control process [2-4]. In the lumen of endoplasmic reticulum (ER), one relevant protein quality control mechanism operates, where misfolded proteins are recognized by ER chaperones and some of them are eventually translocated to the cytosol, in the interface with the ER membrane. Finally, the degradation of non-functional proteins can take place by the ubiquitin-proteasome system in a process known as ER-associated degradation (ERAD) [2-4].

The importance of protein quality control mechanisms is evident if it is taken into account that as much as 30% of all nascent polypeptides are misfolded [5,6]. E3 ubiquitin ligases are associated with ribosomes to degrade proteins with aberrant folds, which mean that several proteins can be degraded during translation [7]. Therefore, it is not surprising that several mutants of genes encoding critical proteasome subunits are lethal. Remarkably, accumulation of misfolded proteins is implicated with several human diseases, especially neurodegenerative illnesses that are associated with protein aggregates [8-10].

Proteins that enter the secretory pathway are directed to the ER, where their folding and post-translational modifications occur. However, when the processing capacity of the ER is overwhelmed, misfolded proteins accumulate in this compartment, which triggers a cell defense mechanism known as the unfolded protein response (UPR). The UPR is mediated by the Ire1p, an RNAse, which is activated when misfolded proteins accumulate in the ER lumen. Activated Ire1p removes an inhibitory intron from the *HAC1 *mRNA, which, in turn, is efficiently translated. Hac1p is a transcription factor responsible for activating genes related to ERAD. To accommodate the accumulation of misfolded proteins until their degradation or their homeostatic recovery, the transcription factors Opi1p and Opi3p (*overproducer of inositol 1 and 3 proteins*) are responsible for controlling the expression of genes involved in expansion of the ER membrane, especially genes encoding proteins that are involved in lipid synthesis [11-14].

Three well-characterized ERAD pathways are present in yeast: ERAD-L, -M and -C, depending on the site of the misfolded lesion. Proteins whose misfolded domains are located in the ER lumen are targeted to ERAD-L, whereas proteins with misfolded membrane domains are directed to ERAD-M and proteins with defective domains on the cytoplasmic side of the ER membrane are degraded by the ERAD-C pathway. Therefore, when a protein is misfolded in the ER lumen or membrane, it is transported to the cytoplasm, polyubiquitinated and subsequently degraded by the proteasome (for a review on this process, see [15]).

The ERAD-C pathway is mainly composed by the E3 ubiquitin ligase Doa10p and its associated protein complex. The Doa10p complex is small when compared to the other two ERAD pathway complexes [2]. In addition to Doa10p (the scaffold membrane protein), the Doa10p complex contains Ubc7p (an E2 ubiquitin conjugating enzyme), its anchoring protein Cue1p and the ATPase complex Cdc48, which is composed of the AAA-ATPase Cdc48p, the cofactors Ufd1p and Npl4p and the complex anchorage protein Ubx2p [2].

Some studies describe a post-ER system of protein quality control, which would occur at the Golgi compartment. This system was suggested to be used in addition to the ERAD pathway upon saturation of the ERAD system by misfolded proteins [16,17]. Only recently, Wang and Ng (2010) characterized a substrate dependent on post-ER Golgi quality control, the protein Wsc1p, which is a transmembrane protein that functions as a sensor of plasma membrane/cell wall integrity [18]. Thus, the description of this quality control process and determination of its specific substrates represented a breakthrough since a novel biological function, *i.e*. degradation of proteins, was revealed.

Here, we show that Pof1p, a protein that was recently reported as a filamentation promoter protein [19], is an ATPase that is likely involved in the protein degradation pathway. The expression of *POF1 *gene was able to suppress the sensitivity of *Δpct1 *strain (mutant for a phosphocholine cytidylyltransferase enzyme) to heat shock; however, the Pof1p enzyme possesses no cytidylyltransferase activity but does have ATPase activity. Some studies have related membrane lipid biosynthesis with the ERAD pathway ([20]; reviewed by [21]) and wide-scale studies of protein-protein physical interactions found Pof1p in complex with Doa10p [22], Ubc7p (Database of Interacting Proteins (DIP), 2010) and Nas2p [23]. Doa10p and Ubc7p are components of the ERAD-C pathway [1], and Nas2p is a protein involved in proteasome assembly [24]. Taken together, the data suggest that the biological function of Pof1p is related to protein quality control.

## Results

**Figure 1:**
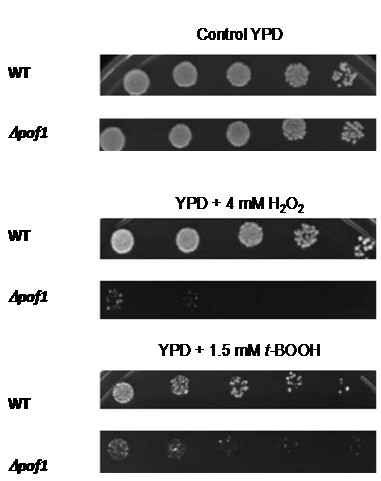
***Δpof1 *cells are sensitive to oxidative stress**. A representative viability assay showing cells exposed to hydrogen peroxide (H_2_O_2_) or *tert-*butyl hydroperoxide (*t*-BOOH) on rich solid media (YPD). The cells (collected at stationary phase) were diluted to OD_600 nm _= 0.2, followed by 4 serial dilutions of 5X. A total of 5 μL of each dilution were spotted on the plates, which were incubated at 30°C for 48 h and photographed.

We were interested to identify deletion mutant strains for genes with unknown functions that might be sensitive to oxidative stress. Therefore, several yeast strains were exposed to hydrogen peroxide (H_2_O_2_) or *tert*-butyl hydroperoxide (*t*-BOOH). Among them, *Δpof1 *(*YCL047C *ORF was named *POF1 *due to its involvement in yeast filamentation process [19]) was highly sensitive to these oxidants (Figure 1).

**Figure 2:**
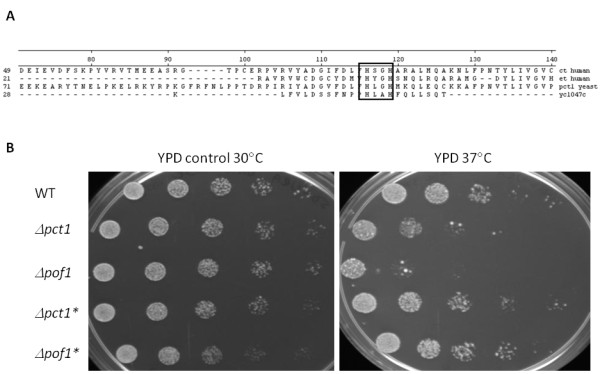
***POF1 *and *PCT1 *sequences and functional analyses**. (A) Clustal W (Megalign software) primary sequence alignment of the cytidylyltransferase family. The conserved motif HxxH is enclosed in the box. Ct human = choline cytidylyltransferase from humans (gi 166214967); et human = ethanolamine cytidylyltransferase from humans (gi 1817548); pct1 yeast = phosphocholine cytidylyltransferase from *S. cerevisiae *(gi 1323361)*; *ycl047c = Pof1p (gi 6319802). (B) Complementation assays. The cells were exposed to heat shock (37°C) or control conditions (30°C) for 17 h, serially diluted and spotted on YPD plates, which were incubated for 48 h at 30°C and photographed. The asterisk denotes cells transformed with the plasmid pYES-TOPO+*POF1 *for overexpression of Pof1.

To get insights on the involvement of Pof1 in the antioxidant cell response, a series of bioinformatics analysis were performed (Protein Information Resource (PIR) site, the UniProt Consortium http://pir.georgetown.edu/cgi-bin/ipcEntry?id=S19376, and the Munich Information Center for Protein Sequences (MIPS) site http://mips.helmholtz-muenchen.de/genre/proj/yeast/searchEntryAction.do?text=YCL047C, indicating that the *POF1 *gene may belong to the cytidylyltransferase family. Therefore, the primary sequence of *POF1 *was aligned with the amino acid sequence of the most studied phosphocholine cytidylyltransferase protein in yeast, *PCT1*, the rate-limiting enzyme in the phosphatidylcholine synthesis pathway, which is a major membrane lipid component. Also, human isoforms of choline (ct human) or ethanolamine (et human) cytidylyltransferases amino acid sequences were aligned with *POF1 *(Figure 2A). Although the overall similarity among sequences was low (around 10%), the conserved motif HxxH [25], which is characteristic of the active site of the cytidylyltransferase family, was present in the predicted primary sequence of *POF1*.

Accordingly, to investigate the hypothesis that Pof1p is a cytidylyltransferase, the biological complementation assay of the *PCT1 *mutant strain was performed by overexpressing *POF1 *in cells challenged with heat shock stress because *Δpct1 *is sensitive to this stress [26]. Overexpression of *POF1 *was able to reverse the heat shock sensitivity of the *Δpct1 *strain (Figure 2B), suggesting that Pof1p and Pct1p share a common function. Indeed, as *Δpct1 *cells, the *Δpof1 *strain was highly sensitive to heat shock. Moreover, overexpression of *POF1 *also partially rescued the wild type phenotype in *Δpof1 *strain.

**Figure 3:**
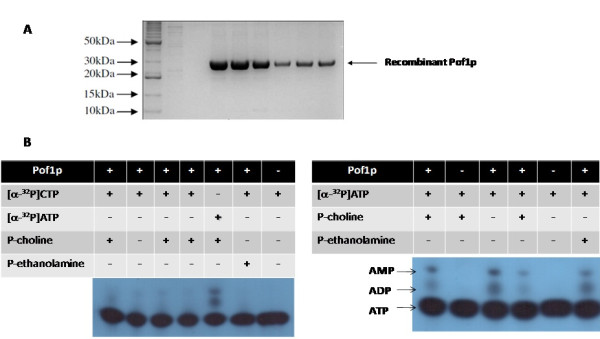
**Pof1p purification and activity analyses**. (A) SDS-PAGE showing the purification of recombinant Pof1p obtained through metal affinity chromatography. Lane 1: molecular weight standard; subsequent lanes were different fractions obtained during the elution process. (B) Thin layer chromatography analyses to observe Pof1p ATP transferase activity; the controls were included to assay for alterations in CTP and ATP. See the Materials and Methods section for details.

Pure, recombinant Pof1p was obtained in the soluble fraction (Figure 3A), and Pof1p was assayed for phosphocholine or phosphoethanolamine cytidylyltransferase activities. Intriguingly, *POF1 *did not hydrolyse CTP as analyzed by thin layer chromatography (TLC), but instead it displayed ATPase activity (Figure 3B). The ATPase activity was independent of the presence of phospholipid precursors in the reaction media, indicating that Pof1p was not interacting with these substrates, at least when hydrolyzing ATP. The reaction products were also analyzed by mass spectrometry, but no CDP-choline or CDP-ethanolamine could be detected (data not shown).

**Figure 4:**
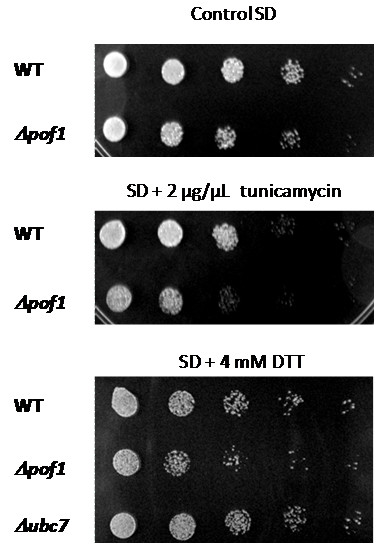
***Δpof1 *cells are sensitive to protein unfolding**. The cells from the stationary phase of growth were diluted to OD_600 nm _= 0.2, followed by 4 serial dilutions of 5X. A total of 5 μL of each dilution were spotted on SD complete media plates containing no unfolding agent, DTT or tunicamycin. The plates were incubated at 30°C for 48 h and photographed.

**Figure 5:**
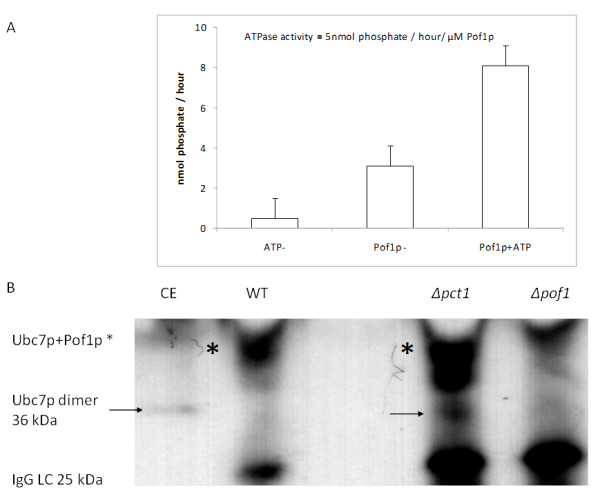
**ATPase activity of Pof1p and its physical interaction with Ubc7 (ubiquitin conjugase 7) protein**. (A) Hydrolysis of ATP as measured by the PiPer™ Phosphate Assay Kit (Invitrogen). (-) ATP represents the result of the reaction in the absence of ATP; (-) *POF1 *represents the result of the spontaneous ATP hydrolysis in the absence of Pof1p; the lane labeled "Pof1p + ATP" contains the complete reaction. The assays were performed at 37°C for 1 h. (B) Western blot analyses of Ubc7p using the commercial antibody Ube2G2 (Abcam). The fractions were obtained from *co-immunoprecipitation *assays using Pof1p polyclonal antibody from the following protein extract: WT = wild type; *Δpct1 *and *Δpof1*. The asterisk shows the Pof1p-Ubc7p complex. CE = total soluble wild type cell extract; IgG LC = IgG light chain. The arrow points Ubc7p dimer.

Since the ability of Pof1p to complement Pct1p function during heat shock is not related to CDP-choline activity, the hypothesis that Pof1p participates in some protein quality control was tested. Cells were submitted to ER stress, by exposing them to high concentrations of dithiothreitol (DTT) and tunicamycin (a protein glycosylation inhibitor). Both agents are well known to provoke accumulation of unfolded proteins in the ER. *Δpof1 *cells displayed higher sensitivity to ER stress agents than wild-type cells and *Δubc7 *cells (mutant strain which lacks *UBC7 *gene which encodes ubiquitin conjugating enzyme involved in ERAD, a control cell line [27]) (Figure 4), suggesting that Pof1p is involved in UPR. Besides, Pof1p presented an ATPase-specific activity of 5 nmol of released phosphate per hour per μM enzyme (Figure 5A). This activity is comparable to the ATPase activity of the ERAD-associated protein Kar2p (approximately 4 nmol of phosphate release per hour per μM of Kar2p in complex with both the nucleotide exchanger Lhs1p and the DnaJ-like partner) [28], indicating that this enzymatic process is relevant *in vivo*.

Interestingly, using the bioinformatics tool PIPE 2 (Protein-Protein Interaction Prediction Engine, freely available at http://pipe.cgmlab.org/), with the default cutoff of 0.06 (sensitivity = 57% and specificity = 89%), we could predict an interaction between the Kar2p ATPase and Pof1p [29]. At a lower cutoff of 0.04 (sensitivity = 70% and specificity = 83%), an interaction between Pof1p and Cdc48p was predicted, which is the ATPase present in all types of ERAD pathways [2,29]. As a positive control, Pof1p and Kss1p interaction was predicted using the default cutoff of 0.06. This is in agreement with experimental data showing through transcriptome data that this mitogen-activated protein kinase (MAPK) (involved in signal transduction pathways that control filamentous growth and pheromone response) interacted with *POF1 *[19]. As a negative control, the ATPase from vacuole VMA10 was not predicted to be an interacting partner with Pof1p, even using a lower cutoff of 0.01 (sensitivity = 92% and specificity = 47%).

To validate these *in silico *protein-protein interactions predictions, anti-Pof1p rabbit polyclonal antibodies were produced. The interactions between Pof1p with Doa10p and with Ubc7p, two components of the ERAD pathway, were investigated, since these complexes were previously described [22]. The physical interaction between Nas2p and Pof1p could not be investigated because there is no commercially available Nas2p antibody. Doa10p and Pof1p did not co-immunoprecipitate under the cell growth conditions tested (data not shown); however, we did observe a physical interaction between Ubc7p and Pof1p *in vivo *(Figure 5B). The migration of the band relative to the Ubc7p and Pof1p corresponded to the sum of the molecular mass of the two proteins. Since *Western blot *was performed in denaturing conditions (after SDS-PAGE) the band depicted with asterisk might be observed due to the formation of a mixed disulfide bond between Pof1p and Ubc7p. Pof1p possesses six cysteine residues. Probably the concentrations of DTT (1 mM) employed were too low to reduce the mixed disulfide between Pof1p and Ubc7p.

**Figure 6:**
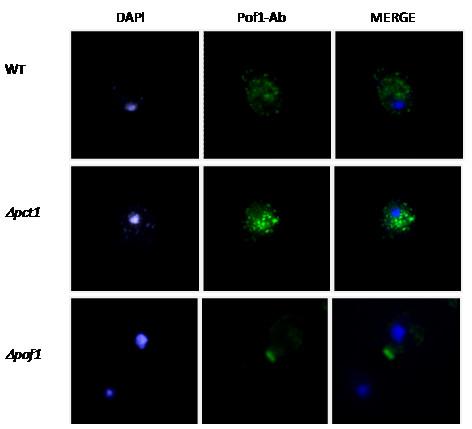
**Immunocytochemistry assays to study Pof1 protein cell localization and distribution**. The *POF1 *null cells were used as a negative control to establish antibody background levels.

Taking advantage of the anti-Pof1p antibody, the Pof1p sub-cellular distribution was studied. A punctuated Pof1p distribution in was observed in wild-type cells (Figure 6), which was more evident in *Δpct1 *cells. This is in agreement with higher protein expression of Pof1p in *Δpct1 *cells, which was also observed by *Western blotting *(data not shown), suggesting a compensatory response. Based on previous immunocytochemistry studies [30], we speculate that Pof1p localizes to the Golgi compartment.

## Discussion

The first suggestion that the *POF1 *gene was related to the protein quality control response arose from wide-scale studies about the relationship between the ERAD and UPR systems [20]. Indeed, mRNA levels of *POF1 *gene were significantly increased in cells that were treated with ER stress agents (DTT and tunicamycin), and this induction was dependent on both Ire1p and Hac1p. In addition, a proteasome inhibitor (PS-341) provoked a four-fold induction of *POF1 *gene expression [31].

Furthermore, the expression of *POF1 *gene is repressed in the *Δopi1 *strain [20], suggesting an involvement of Pof1p with membrane and protein metabolism. The viability data presented here are in agreement with this idea, especially when considering the fact that all stressful conditions tested (oxidative, heat shock, and ER stress in Figures 1, 2 and 4) are well known to provoke protein misfolding. Yet, oxidative stress and heat shock (Figures 1 and 2) caused the most severe phenotypes in *Δpof1 *cells, which is likely due to the fact that these stresses damage both membrane and protein homeostasis [32,33]. The fact that *POF1 *overexpression was able to complement the function of *PCT1 *in *Δpct1 *cells during heat shock (Figure 2) and its expression levels by Opi1p [20] suggests the involvement of Pof1p in membrane lipid metabolism. In addition, the levels of Pof1p are augmented in *Δpct1 *cells (Figure 6 and western blot analyses - data not shown), which indicated that Pof1p might at least partially backing up Pct1p.

However, the molecular function of Pof1p could not be directly related to membrane lipid synthesis although the protein displayed ATPase activity (Figure 3). Pof1p ATPase activity was efficient enough to occur *in vivo*, and its specific activity was comparable to Kar2p, an Hsp70 chaperone from the ER that reaches its maximum activity (approximately 4 nmol of released phosphate per hour per μM of Kar2p) when in complex with co-chaperones Sec63p (a soluble protein that contains a J-domain) and Lhs1p [28]. Pof1p ATPase activity was also comparable with p97, the mammal homolog of yeast Cdc48p, which is the main ERAD ATPase [34,35]. As indicated by PIPE 2 bioinformatics analyses Pof1p is predicted to interact with others proteins involved in ERAD, such as Kar2p and Cdc48p.

In addition to viability and activity results indicating that Pof1p is involved in protein quality control, protein-protein interactions studies in wide-genome scale indicated the participation of Pof1p as a component of the ubiquitin-proteasome pathway. Hesselberth *et al. *(2006) described the Doa10p-Pof1p complex using protein microarray technology, whereas The DIP site and *Genemania *Fast Gene Function Predictions tool (September 2^nd^, 2010 database update) reported the Ubc7p-Pof1p interaction. Under our growth conditions of stationary growth phase and galactose-containing medium, we did not observe Doa10p-Pof1p co-immunoprecipitation (data not shown); however, under the same growth conditions, we detected an Ubc7p-Pof1p interaction (Figure 5B).

Still taking advantage of a polyclonal Pof1p antibody produced in this study, a punctuated Pof1p cell distribution was observed (Figure 6) that is very similar to proteins localized in the Golgi compartment [30]. Although these results are preliminary, the immunocytochemical data clearly showed that Pof1p is not uniformly distributed in the cytoplasm and does not co-localize with the nucleus or mitochondria where DNA is stained with DAPI (see merged figure, Figure 6). Since ER protein distribution is expected to be perinuclear, Pof1b probably was not located in this organelle.

The post-ER Golgi protein quality control pathway has already been reported, and at least one specific substrate of this system has been characterized [36]. Taken together, the results suggest that Pof1p is an ATPase that interacts with the ubiquitin conjugating protein (an E2) Ubc7p and protects cells from accumulating misfolded proteins caused by oxidative, heat, reductive or chemically (tunicamycin) stressful conditions. A possible explanation for the functional relationship between Pct1p and Pof1p could be due to the participation of Pof1p in protein quality control. For instance, the autophagy system controls the turnover of the majority of stable proteins and coordinates degradation through the engulfment of these polypeptides into a double-lipid bilayer - the autophagosome - which fuses with a lysosome/vacuole in which degradation occurs [37]. Given that *Δpct1 *cells have deficient membrane lipid turnover [38], which probably results in lower membrane repositioning during autophagy, the ER expansion would be impaired. In this situation, an increase in Pof1p levels, together with several other proteins, would improve the proteasomal degradation process.

The results presented here, together with previously published data, allow us to speculate that Pof1p is part of the ERAD pathway as an E3 ubiquitin ligase. Pof1p may be involved in substrate recognition during ubiquitin marking because it interacts physically with an E2 ubiquitin conjugating enzyme, Ubc7p, and it is important in the unfolded protein response. *Δpof1 *cells were more sensitive to reductive stress than the *Δubc7 *cells (cells in which Ubc7p is absent), this last a well-characterized protein that participates in the ERAD-C pathway. A possible substrate would be the MAP kinase molecule Kss1p, which interacts physically with Pof1p [19]. As mentioned above, Kss1p is a kinase involved in the control of filamentous growth and the pheromone response. Fasolo *et al. *(2011) observed that *Δpof1 *cells are defective in invasive growth and pseudohyphal growth. We hypothesize that the phenotype observed in *Δpof1 *cells is due to the absence of stability regulation of Kss1p exerted by Pof1p.

Therefore, the results described here showed that a protein involved in the yeast-to-hyphal transition [19] possesses ATPase activity and is important in the response of yeast to various stresses. A study on gene expression modulation during yeast filamentous-form growth showed an enriched number of genes involved in protein quality control, such as N-linked glycosylation, ubiquitin-dependent protein catabolism and ER to Golgi transport. Moreover, this study pinpointed the 26S proteasome as an important component in the regulation of *S. cerevisiae *filamentous growth [39]. The yeast-to-hyphal transition is a response of several fungi to stressful conditions. For the majority of pathogenic fungi, this transformation is an essential step in their infectious process, and modifications in plasma membrane and cell wall constituents have been implicated [40,41]. The mechanisms that trigger the transition to filamentous growth in *S. cerevisiae *are associated with carbon or nitrogen stresses [39,42]. The interplay between the filamentation process and protein quality control may be an important feature that deserves to be further investigated.

## Conclusions

This study characterized the molecular function of Pof1p as an ATPase involved in protein quality control. Pof1p was important to yeast defense against oxidative, heat shock and chemically induced stress. Several protein quality control components are still poorly described, despite their importance in neurological diseases. The molecular characterization of the components in yeast can be useful to understand the function of conserved human proteins.

## Methods

Chemicals: t-BOOH, tunicamycin and DTT were purchased from Sigma Chemical Company (St. Louis, MO, USA). The other chemicals used were analytical grade or better. H_2_O_2 _(30%) was obtained from Merck.

Yeast strains and growth conditions: The yeast strains used here were obtained from the Yeast Deletion Clones repository (Invitrogen - Carlsbad, CA, USA). The wild-type strain was BY4741 (*Mata; his3Δ1; leu2Δ0; met15Δ0; ura3Δ0*); the mutant strains were *Δpof1 *(*Mata; his3Δ1; leu2Δ0; met15Δ0; ura3Δ0; YCL047C::kanMX4*); *Δpct1 *(*Mata; his3Δ1; leu2Δ0; met15Δ0; ura3Δ0; YGR202C::kanMX4*); *Δubc7 *(*Mata; his3Δ1; leu2Δ0; met15Δ0; ura3Δ0; YMR022W::kanMX4*). The yeast cells were grown in YPD (1% yeast extract, 2% peptone and 2% dextrose), YPGAL (1% yeast extract, 2% peptone and 2% galactose) or complete synthetic medium (0.17% yeast nitrogen base (YNB), 0.5% ammonium sulfate, all required amino acids plus 2% glucose). SD = synthetic dextrose medium. For most analyses, when yeast strains were grown on glucose or galactose, the cells were harvested by centrifugation at stationary phase, which corresponds to an OD_600 nm _between 2.0 and 5.0.

Viability assays: The tolerance of yeast cells to H_2_O_2 _or to *t*-BOOH was determined by the spot test, as described below. Inoculates were obtained from cells that were grown overnight in YPD or complete synthetic media with 2% glucose (indicated in the figures). Inoculates were diluted to OD_600 nm _= 0.2, and yeasts were grown until cell density reached stationary phase (around 16 h). Finally, the cell cultures were diluted again to OD_600 nm _= 0.2, and then four subsequent 1:5 dilutions of these cell suspensions were performed. A 5 μL droplet of each dilution was plated onto YPD or complete synthetic medium (SD) plus agar with the stress agent. Peroxides were added to plates at the concentrations indicated in the figures. DTT or tunicamycin was spread onto the plates just before use. To test cell viability under heat shock conditions, the strains were grown until cell density reached OD_600 nm _= 0.8, and they were divided into two aliquots, which were incubated at 30°C (control) or 37°C. The serial dilutions (starting from OD_600 nm _= 0.2) were spotted onto YPD agar plates, and the plates were incubated for 48 h at 30°C.

Construction of yeast overexpression vector pYES-TOPO + *POF1*: The coding region of *POF1 *gene was cloned from yeast genomic DNA using the following specific primers: *POF1 *forward 5'TGCTGTCACATATGAAGAAGAC and *POF1 *reverse 5'TAAACGGATCCTCAATCAAATATTG, which contain *NdeI *or *BamHI *restriction enzyme sites adaptors, respectively (underlined sequences). This PCR-isolated DNA fragment was purified with the GFX PCR DNA and Gel Band Purification kit (GE Healthcare, Uppsala, Sweden) and ligated into the pYES-TOPO backbone to form pYES-TOPO + *POF1 *for yeast expression (controlled by *GAL1 *promoter) and into the pET15b vector to generate pET15b + *POF1 *for bacterial expression (controlled by T7 promoter). The *POF1 *gene was added to pYES2.1-TOPO TA (Invitrogen) reaction media according to the manufacturer. The ligation product was transformed into *Escherichia coli *DH5α bacteria strain by electroporation. The transformed clones were grown in LB + ampicillin (100 μg/mL), and the plasmids were isolated with the Illustra plasmidPrep Mini Spin Kit (GE Healthcare). The clones were analyzed for proper directionality of the insertion by PCR using the TOPO *GAL *forward primer and the *POF1 *reverse primer. The correct plasmids were sequenced and transformed into the respective yeast strains by electroporation [43].

Heterologous expression and purification of recombinant Pof1p: Recombinant Pof1p, which possesses an N-terminal His-tag, was expressed in the *E. coli *BL21 (*DE3*) strain that was transformed with the pET15b-Pof1p plasmid (the *POF1 *coding region was cloned into the expression vector pET15b from Novagen using the *NdeI *and *BamHI *restriction sites). The cells were cultured (50 mL) overnight in LB + ampicillin (100 μg/mL), transferred to 1 L of fresh LB + ampicillin medium and cultured further until the OD_600 nm _reached 0.6-0.8. IPTG was added to a final concentration of 1 mM. After 3 h of incubation at 37°C, the cells were harvested by centrifugation. The pellet was washed and suspended in the start buffer composed of 50 mM Tris-HCl (pH 7.4), 100 mM NaCl and 20 mM imidazole. The cells were sonicated twice for 45 s (40% amplitude), followed by 30 s on ice between sonications using a Branson Cell Disruptor. The cell extracts were kept on ice during streptomycin sulfate treatment (1% for 20 min), and the suspension was centrifuged at 16,000 g for 30 min to remove nucleic acid precipitates and cell debris. Finally, the extracts were applied to a Hi-trap nickel-affinity column (Life Technologies). The conditions for protein purification were optimized using the gradient procedure for imidazole concentration described by the manufacturer.

Thin Layer Chromatography (TLC) analyses: The assays were performed as previously described [44]. Briefly, the reaction media contained 50 mM Tris-HCl (pH 7.4), 100 mM NaCl, 10 mM MgCl_2_, 20 μM phosphatidylcholine:oleate vesicles, 10 mM DTT, 1.5 mM phosphocholine (or 2 mM phosphoethanolamine), 1 μg/μL (20 μM) Pof1p and 200 μCi/μmol of [α-^32^P]CTP or [α-^32^P]ATP. The reactions were incubated at 37°C overnight in the presence of [α-^32^P]CTP or 2 h in the presence of [α-^32^P]ATP. Controls were subjected to the same conditions in the absence of Pof1p. The reactions were analyzed by TLC at room temperature using silica gel plates (Merck) with a solvent system composed of ethanol/NH_4_OH (1:1). The plates were autoradiographed, and the resulting bands were compared with [α-^32^P]CTP or [α-^32^P]ATP without any incubation or addition of enzyme.

ATPase activity. The reactions containing 1 mM ATP, 1 μM Pof1p, 5 mM MgCl_2 _and 100 mM Tris-HCl (pH 7.5) were incubated at 37°C for 1 h. Subsequently, the reactions were boiled for 5 min and centrifuged for 10 min at 16,000 g. The P_i_Per Phosphate assay mix was added to the supernatant according to the manufacturer's instructions (Molecular Probes - Invitrogen). The reactions were incubated at 37°C for an additional 1 h in the dark. The absorbance of resorufin, the Amplex Red reagent reaction product, was detected by its absorbance at 565 nm. A calibration curve with known concentrations of inorganic phosphate was used to quantify the P_i _released during the ATPase reactions. Controls without Pof1p and without substrate (ATP) were subjected to the same conditions.

Co-immunoprecipitation assays: Wild type, *Δpct1 *and *Δpof1 *cells were grown until stationary phase in synthetic galactose complete medium. The cells were centrifuged and washed with 1X phosphate-buffered saline (PBS). The cells were lysed using glass beads in lysis buffer (50 mM Hepes (pH 7.5), 5 mM EDTA, 150 mM NaCl, 300 mM KCl, 1% Triton X-100, 2 mM PMSF, 5% glycerol and 20 mM β-mercaptoethanol). The insoluble fraction was separated by centrifugation at 16,000 g for 30 min and 4°C. The soluble fraction was incubated with a Dynabead-anti-Pof1p complex overnight at room temperature under gentle agitation. The complexed proteins were washed three times using the washing buffer provided by the Dynabeads Protein G kit (Invitrogen), and the samples were eluted using 20 μL of elution buffer (provided in the kit), incubated for 10 min at 70°C in 10 μL of 5X protein SDS-PAGE loading buffer and 1 mM DTT (recommended 10 mM). One-third of each sample was subjected to western blot analyses.

Western blot analyses: Immunoblot analyses were performed using rabbit polyclonal antibodies against Pof1p produced in this study by immunization with pure recombinant Pof1p. The commercial antibodies from Abcam were used to study Doa10p (mouse monoclonal antibody to MARCH6 (ab56594)) and Ubc7p (rabbit polyclonal antibody to Ube2G2 (ab97279)). Proteins were transferred to nitrocellulose, and the processing of nitrocellulose blots was performed using the BioRad system. The HRP and luminol-based reagent from ECL (Amersham GE Healthcare) was used as a detection system. The membranes were autoradiographed using Amersham Hyperfilm and photo-documented.

## Competing interests

The authors declare that they have no competing interests.

## Authors' contributions

IMC, THTN performed the majority of the experiments. SM and FMP carried out TLC and mass spectrometry analyses. MD and RMPN executed the antibody production and immunocytochemistry studies. GM and LESN have made substantial contributions to conception and design, analysis and interpretation of data. All authors have been involved in drafting the manuscript or revising it critically for important intellectual content.
